# Supplementary material for: Effects of Methionine and Glutathione on Acute Ototoxicity Induced by Amikacin and Furosemide in an Animal Model of Hearing Threshold Decrease
Source: Biomedicines. 2025 Jun 15;13(6):1476. doi: 10.3390/biomedicines13061476 (PMC12191240; doi:10.3390/biomedicines13061476)
Supplement: Supplementary file 1 [file biomedicines-13-01476-s001.zip › biomedicines-3606642-supplementary.pdf]

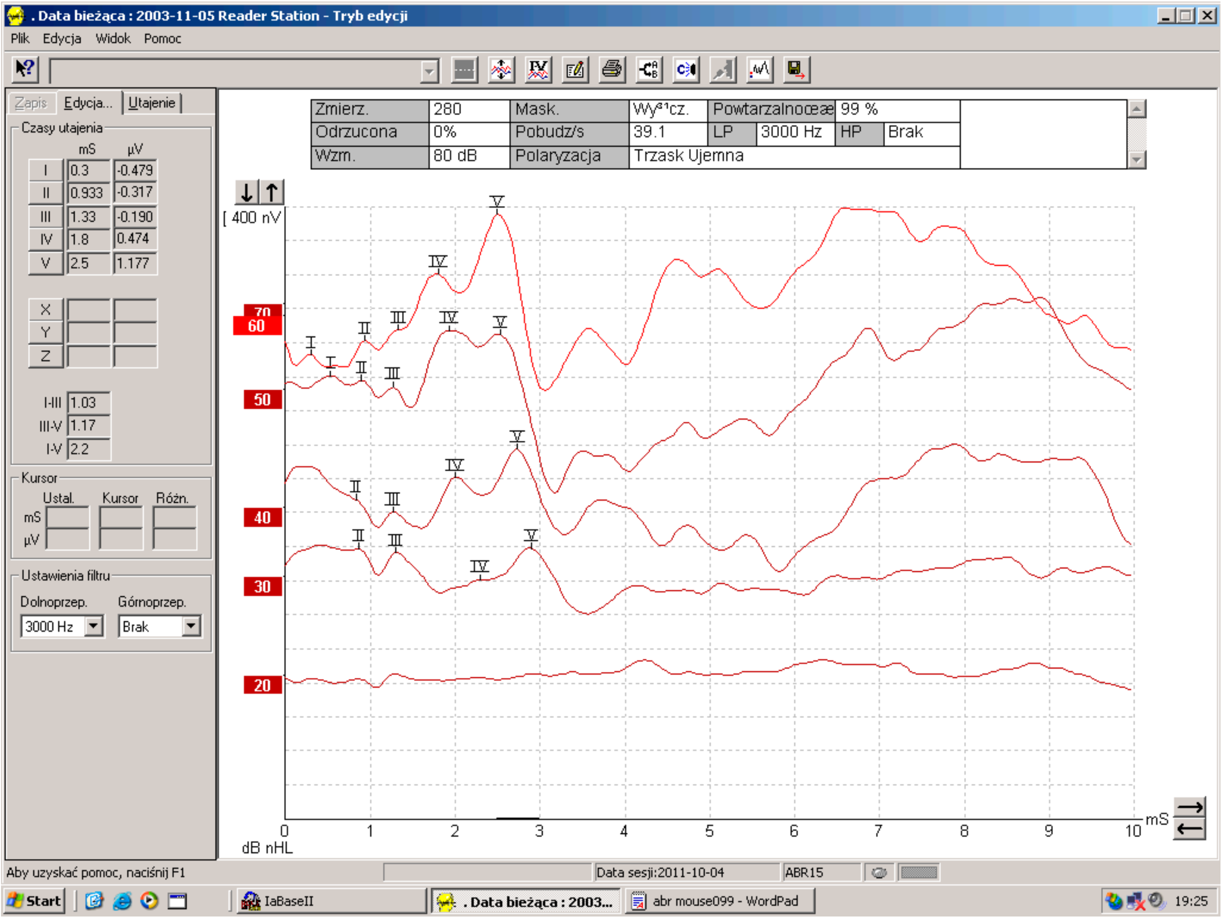

Figure S1. The ABR wave V analysis (baseline threshold). Print screen from the Interacoustics Eclipse EP15 unit (Middelfart, Denmark) that measured and collected auditory brainstem responses (ABRs) in mice.
